# Atopic dermatitis and chronic kidney disease: a bidirectional Mendelian randomization study

**DOI:** 10.3389/fmed.2023.1180596

**Published:** 2023-06-27

**Authors:** Han Zhang, Shuai Yuan, Yong Li, Doudou Li, Zengli Yu, Lidan Hu, Xue Li, Yuming Wang, Susanna C. Larsson

**Affiliations:** ^1^Henan Provincial People’s Hospital, Zhengzhou University, Zhengzhou, China; ^2^College of Public Health, Zhengzhou University, Zhengzhou, China; ^3^Unit of Cardiovascular and Nutritional Epidemiology, Institute of Environmental Medicine, Karolinska Institute, Solna, Stockholm, Sweden; ^4^Department of Biometry, Institute of Genetic Epidemiology, Epidemiology and Medical Bioinformatics, Faculty of Medicine and Medical Center - University of Freiburg, Freiburg, Germany; ^5^NHC Key Laboratory of Birth Defects Prevention and Henan Key Laboratory of Population Defects Prevention, Zhengzhou, China; ^6^The Children’s Hospital, Zhejiang University School of Medicine, National Clinical Research Center for Child Health, Hangzhou, China; ^7^Unit of Medical Epidemiology, Department of Surgical Sciences, Uppsala University, Uppsala, Sweden

**Keywords:** atopic dermatitis, chronic kidney disease, Mendelian randomization, MR-PRESSO, bidirectional Mendelian randomization

## Abstract

**Background:**

A bidirectional association between atopic dermatitis and chronic kidney disease (CKD) has been revealed in observational studies, whereas the causality of this association was unclear. We conducted a Mendelian randomization study to determine the bidirectional causal association between atopic dermatitis and CKD.

**Methods:**

Independent genetic instruments associated with atopic dermatitis and CKD at the genome-wide significance level were chosen from corresponding meta-analyses of genome-wide association studies. Summary-level data for atopic dermatitis were obtained from the EAGLE Eczema consortium (30,047 cases and 40,835 controls) and FinnGen consortium (7,024 cases and 198,740 controls). Summary-level data for CKD were derived from CKDGen consortium (64,164 cases and 625,219 controls) and FinnGen consortium (3,902 cases and 212,841 controls). The inverse-variance weighted method was used in the main analysis and supplemented with three sensitivity analyses.

**Results:**

Genetic predisposition to atopic dermatitis was associated with an increased risk of CKD. For a one-unit increase in the prevalence of atopic dermatitis, the odds ratio of CKD was 1.07 (95% confidence interval: 1.01–1.12). In the reverse Mendelian randomization analysis, the odds ratio of atopic dermatitis was 1.14 (95% confidence interval: 1.03–1.26) for a one-unit increase in the prevalence of CKD. The associations persisted in sensitivity analyses and no pleiotropy was detected.

**Conclusion:**

This Mendelian randomization study suggests a bidirectional positive association between atopic dermatitis and CKD.

## Introduction

Chronic kidney disease (CKD) is a common health issue affecting 7–12% of adults worldwide (1). It causes a large disease burden and ranks fourteenth on the list of leading causes of death in the world (2). Renal disease is a common comorbidity in adults with atopic dermatitis (3). In a case–control study with approximately 100,000 participants, individuals with atopic dermatitis were found to have a higher risk of CKD compared to those without atopic dermatitis (4). However, this positive association was not replicated in a cohort study including 335,827 individuals with diabetes mellitus (4). Conflicting and limited evidence, along with possible limitations (e.g., residual confounding and reverse causality) in observational studies, makes the association between atopic dermatitis and CKD uncertain. A clear appraisal of this association may deepen the understanding of CKD’s etiological basis as well as update the prevention strategy for CKD.

Utilizing genetic variants as instrumental variables for exposure (e.g., atopic dermatitis), Mendelian randomization (MR) analysis can strengthen causal inference in an exposure-outcome association by minimizing residual confounding and reverse causality (5). Since genetic variants are randomly assorted at conception and therefore generally unassociated with confounders, like environmental or self-adopted factors, the MR approach can reduce residual confounding. In addition, the method can diminish reverse causation as genetic variants cannot be modified by the onset or progression of the disease.

Here, we conducted an MR study to examine the impact of atopic dermatitis on CKD risk. Given that pruritus caused by skin diseases is a common symptom in CKD patients (6–8), we performed a reverse MR analysis to assess the influence of CKD on the risk of atopic dermatitis.

## Methods

### Study design

**Figure 1:**
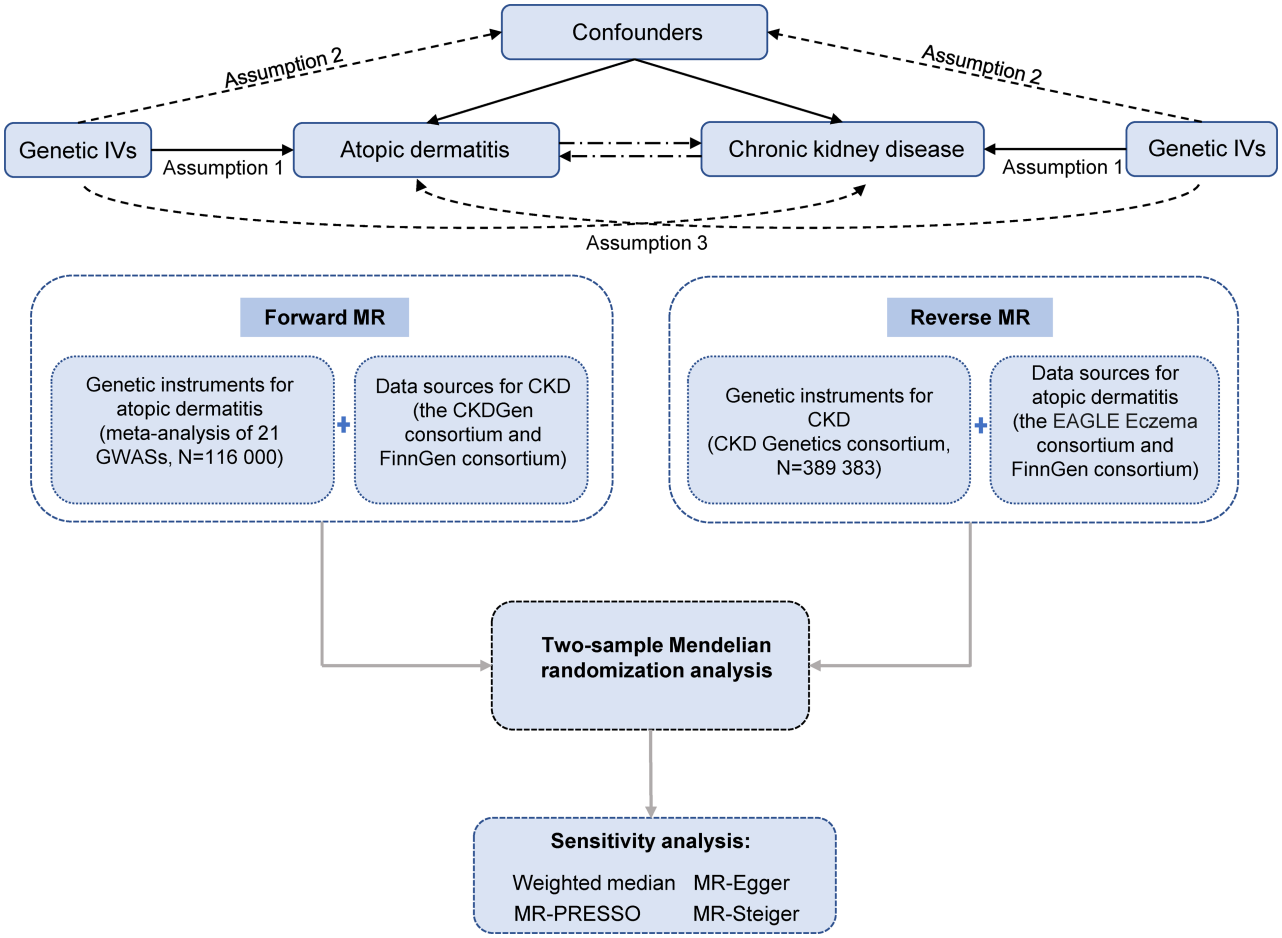
Study design overview. IVs, instrumental variables; MR, Mendelian randomization; CKD, chronic kidney disease; EAGLE, EArly Genetics and Lifecourse Epidemiology. Assumption 1 indicates that the genetic variants proposed as instrumental variables should be robustly associated with the exposure. Assumption 2 indicates that instrumental variables should not be associated with potential confounders. Assumption 3 indicates that instrumental variables should affect the risk of the outcome merely through the risk factor, not *via* alternative pathways.

The MR study was based on publicly available summary-level data from corresponding consortia (Table 1). The study design overview is presented in Figure 1. All included studies had been approved by relevant ethical review boards and participants had given informed consent.

**Table 1:** Information on used consortia.

Exposure or outcome	Participants included in analysis	Adjustments
**Instrument selection**		
Chronic kidney disease	64,164 cases and 625,219 controls of multi-ancestries	Age, sex, study site, genetic principal components, relatedness, and other study-specific features
Atopic dermatitis	Up to 21,000 cases and 95,000 controls of European ancestry	Not reported
**Outcome data sources**		
CKDGen consortium	64,164 chronic kidney disease cases and 625,219 controls of multi-ancestries	Age, sex, study site, genetic principal components, relatedness and other study-specific features
FinnGen consortium (CKD)	3,902 chronic kidney disease cases and 212,841 controls of European ancestry	Age, sex and up to 20 genetic principal components
Available GWAS (MN)	2,150 MN cases and 5,829 controls of European ancestry	Cohort-specific significant principal components
FinnGen consortium (diabetic nephropathy)	3,283 Cases and 210,463 controls of European ancestry	Age, sex and up to 20 genetic principal components
FinnGen consortium (glomerular nephritis)	4,613 Cases and 214,179 controls of European ancestry	Age, sex and up to 20 genetic principal components
FinnGen consortium (hypertensive nephropathy)	468 Cases and 162837controls of European ancestry	Age, sex and up to 20 genetic principal components
FinnGen consortium (nephrotic syndrome)	480 Cases and 214,619 controls of European ancestry	Age, sex and up to 20 genetic principal components
EAGLE consortium	30,047 atopic dermatitis cases and 40,835 controls of European ancestry (after exclusion of 23 and Me)	Not reported
FinnGen consortium (atopic dermatitis)	7,024 atopic dermatitis cases and 198,740 controls of European ancestry	Age, sex and up to 20 genetic principal components

CKD, chronic kidney disease.

### Genetic instrument selection for atopic dermatitis

Twenty-one single nucleotide polymorphisms (SNPs) associated with atopic dermatitis at the genome-wide significance level (*p* < 5 × 10^−8^) were identified from a meta-analysis of 21 genome-wide association studies (GWASs) including up to 21,000 cases and 95,000 controls of European ancestry (9). Associations for identified SNPs with atopic dermatitis were further replicated in a meta-analysis of 18 GWASs incorporating 32,059 cases and 228,628 controls (9). These SNPs were free of linkage disequilibrium (*r^2^* < 0.01 and clump window >10,000 kb) and used as instrumental variables for atopic dermatitis. Detailed information on genetic instruments is displayed in Supplementary Table S1.

### Genetic instrument selection for CKD

SNPs associated with CKD at the genome-wide significance level (*p* < 5 × 10^−8^) were selected from CKD Genetics consortium including 64,164 cases and 625,219 controls of multi-ancestries (10). We calculated linkage disequilibrium among these SNPs using 1,000 Genomes European panel as the reference population (11). Thirty-seven independent SNPs (*r^2^* < 0.01 and clump window >10,000 kb) were identified as instrumental variables for CKD. Detailed information on genetic instruments is displayed in Supplementary Table S2.

### Data sources for CKD and subsets

Summary-level data for CKD were obtained from the CKDGen consortium and FinnGen consortium. CKDGen includes data from more than 120 GWASs with 64,164 cases and 625,219 controls of multi-ancestries (10). CKD cases were defined by the estimated glomerular filtration rate on creatinine <60 mL/min per 1.73 m^2^. The R5 release of genome-wide analysis on CKD in FinnGen consortium was used, which includes 3,902 cases and 212,841 controls of European ancestry (12). Cases of CKD in FinnGen were defined by hospital discharge and cause of death code N18 of International Classification of Disease-10 (ICD-10) and 585 of ICD-9. To evaluate the associations between genetically liability to atopic dermatitis and the risk of different subtypes of CKD, we additionally performed subgroup analyses by using available published GWASs for CKD subsets. The GWAS summary-level data of membranous nephropathy (MN) was derived from an available GWAS conducted in 7979 (2,150 MN cases) European subjects (13). For diabetic nephropathy (3,283 cases and 210,463 controls), glomerular nephritis (4,613 cases and 214,179 controls), hypertensive nephropathy (468 cases and 162,837 controls), and nephrotic syndrome (480 cases and 214,619 controls), their GWAS summary-level data were also obtained from the R5 release of FinnGen consortium (14).

### Data sources for atopic dermatitis

Summary-level data for atopic dermatitis were available from the EAGLE (EArly Genetics and Lifecourse Epidemiology) Eczema consortium and FinnGen consortium. Data from 23andMe were excluded from the EAGLE consortium due to ethical issues, leaving 30,047 cases and 40,835 controls of European ancestry (9). Atopic dermatitis cases were defined by self-reported information, clinical diagnosis, and Hanifin and Rajka criteria (9). The R5 release data of FinnGen consortium includes 7,024 atopic dermatitis cases and 198,740 controls. Cases were ascertained by hospital discharge and cause of death code L20 of ICD-10, 6,918 (with the exclusion of 6,918X) of ICD-9, and 691 of ICD-8.

### Statistical analysis

The genetic correlation between atopic dermatitis and CKD and CKD subsets was calculated using data from the EAGLE Eczema, CKDGen consortia, FinnGen consortium, and available GWAS study. The inverse variance weighted model with random effects was used as the primary statistical method and supplemented with three sensitivity analyses, including weighted median, MR-Egger, and MR-PRESSO methods. Estimates from the primary analysis in different sources were combined using the fixed effects meta-analysis method (for CKD and atopic dermatitis). Assuming that half of the instrument weights are derived from valid instrumental variables, the weighted median method can provide consistent estimates (15). The MR-Egger regression can detect and correct for possible unbalanced pleiotropy albeit usually with underpowered estimates (16). The MR-PRESSO method can detect possible pleiotropic outliers and generates causal estimates after the removal of corresponding outliers (17). We used Cochrane’s Q to assess the heterogeneity among estimates of SNPs in one analysis and the *p* value for the intercept in MR-Egger as an indication of pleiotropy (16). The MR-Steiger analysis was used to test the direction of the potential causal associations (18). All statistical tests were two-sided. The genetic correlation was calculated using *ldsc* software (19, 20). MR analysis was performed using the ‘TwoSampleMR’ (21), ‘MendelianRandomization’ (22), and ‘MR-PRESSO’ (17) packages in R Software 3.6.0. To convert the effect size (β) into the odds ratio (OR), the following formula was used: OR = exp. (β); confidence interval (CI) = exp. (β ± 1.96*SE).

## Results

**Figure 2:**
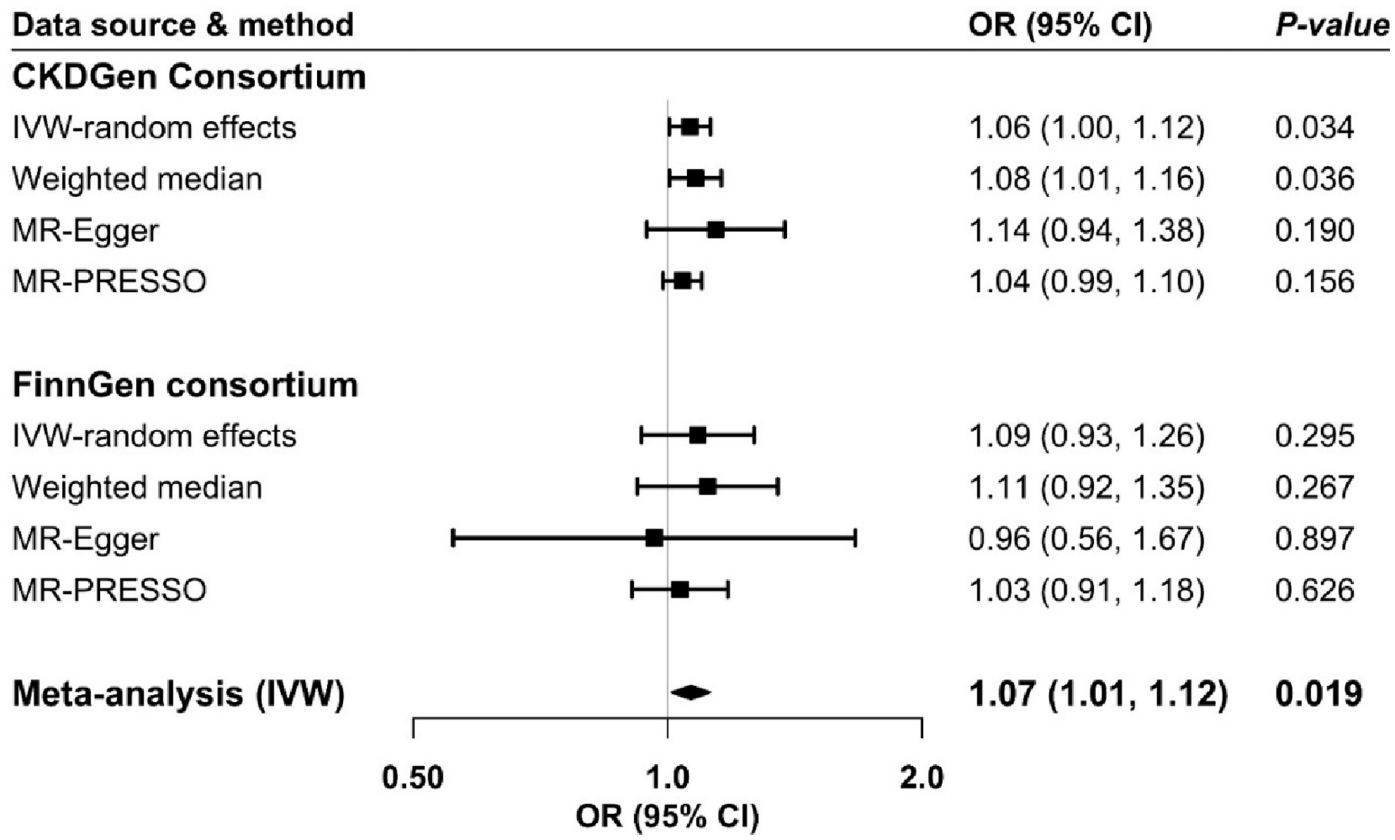
Odds ratio of chronic kidney disease for genetic predisposition to atopic dermatitis. CI, confidence interval; CKD, chronic kidney disease; IVW, inverse variance weighted; OR, odds ratio.

A possible inverse genetic correlation between atopic dermatitis and CKD was observed (*r_g_* = −1.65; *p* = 0.099). One SNP was unavailable in both directional MR analyses in FinnGen and replaced by a proxy SNP (*r*^2^ > 0.8). Genetic predisposition to atopic dermatitis was associated with an increased risk of CKD in the analysis based on CKDGen consortium and in the meta-analysis of CKDGen and FinnGen data (Figure 2). For a one-unit increase in the prevalence of atopic dermatitis, the odds ratio of CKD was 1.06 (95% confidence interval (CI): 1.00, 1.12) and 1.07 (95% CI: 1.01, 1.12) in CKDGen consortium and meta-analysis, respectively. The association persisted in the weighted median analysis based on CKDGen consortium and remained directionally consistent in other sensitivity analyses (Figure 2). We observed mild to moderate heterogeneity in the main analyses, but no pleiotropy was detected by MR-Egger regression (Supplementary Table S3).

In the subgroup analysis for CKD, we found no significant association between genetically liability to atopic dermatitis and the risk of CKD subtypes (all *p* < 0.05; Supplementary Table S4). In addition to hypertensive nephropathy, the effect direction of genetic predisposition to atopic dermatitis on diabetic nephropathy, glomerular nephritis, nephrotic syndrome, and membranous nephropathy kept consistent with the primary analysis for CKD. Although heterogeneity of the used instruments was observed in the analysis for MN, diabetic nephropathy, and glomerular, MR-Egger regression indicated no pleiotropy (Supplementary Table S4). Notably, the statistical power of the analysis for hypertensive nephropathy and nephrotic syndrome may be weak since the cases of these two diseases were smaller.

**Figure 3:**
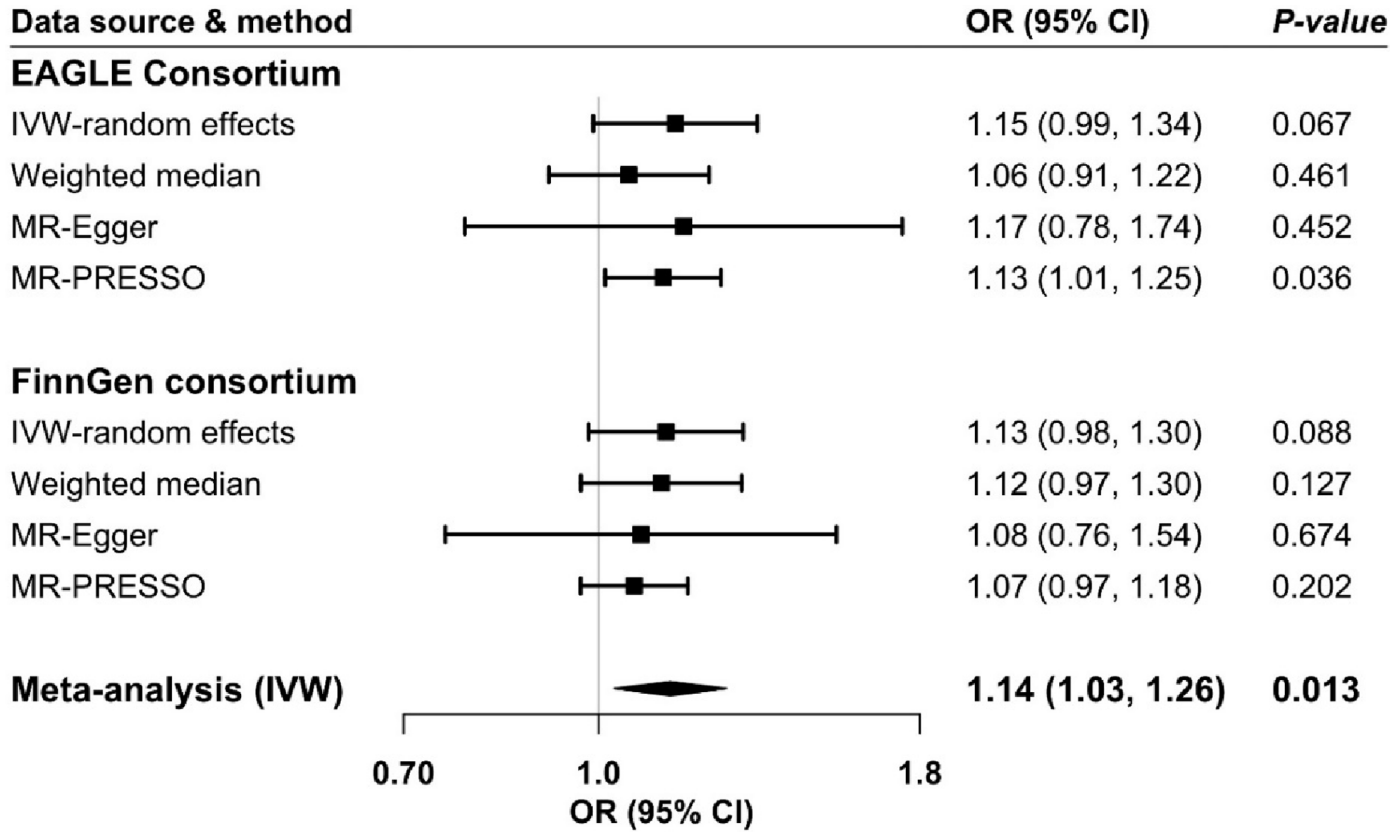
Odds ratio of atopic dermatitis for genetic predisposition to chronic kidney disease. CI, confidence interval; EAGLE, EArly Genetics and Lifecourse Epidemiology; IVW, inverse variance weighted; OR, odds ratio.

Genetic liability to CKD showed a borderline association with atopic dermatitis risk in both analyses based on data from the EAGLE and FinnGen consortium (Figure 3). The association became clear in the meta-analysis of the two sources (Figure 3). The combined odds ratio of atopic dermatitis was 1.14 (95% CI: 1.03, 1.26) for a one-unit increase in the prevalence of CKD. The association was directionally consistent in sensitivity analyses. We detected high heterogeneity in the primary analyses; however, no pleiotropy was observed by MR-Egger (Supplementary Table S3). The association became stronger in the MR-PRESSO analysis based on the EAGLE consortium after the removal of two outliers (Figure 3). Results from the MR-Steiger analysis indicated that the causality of the bidirectional association between atopic dermatitis and CKD was highly likely to be causal (*p* for Steiger analysis <0.05).

## Discussion

Even though atopic dermatitis might be genetically inversely associated with CKD, the MR analysis found a bidirectional positive association between atopic dermatitis and CKD. To the best of our knowledge, this is the first MR study that examined the mutual impacts between atopic dermatitis and CKD.

Studies focusing on the influence of atopic dermatitis on CKD are limited. A matched case–control study of Danish adults found that mortality due to urogenital diseases is more common among individuals with atopic dermatitis compared to those without (23). Another case–control study observed that participants with atopic dermatitis were more likely to have stage 3–5 CKD regardless of the severity of atopic eczema (4). The odds ratio of stage 3–5 CKD ranged from 1.07 to 1.86 for individuals with mild to severe atopic dermatitis (4). However, the positive association between atopic dermatitis and CKD was not found in a cohort study including 335,827 adults with diabetes mellitus (4). The cohort analysis was based on generally young participants with a mean age of 58.5 years and a mean follow-up time was 5.3 years, which might be insufficient to capture differences in risk of incident CKD between those with and without atopic dermatitis (4). In addition, this cohort study used data from patients with diabetes. The features of this special population, such as varying medications (24) and special diets (25), might also influence the association. Our study based on genetic data derived from large consortia suggested a possible positive link between atopic dermatitis and CKD. Cohort studies with a large sample size and long follow-up time and MR studies in different populations are warranted to verify our results.

Underlying mechanisms linking atopic dermatitis to CKD are uncovered, but chronic inflammation appears to be a compelling explanation. Inflammation in patients with atopic dermatitis is thought to be caused by disruption of the epidermal barrier and activation of epidermal inflammatory dendritic and innate lymphoid cells, that attracts and interacts with invading Th2 cells (26). Besides the Th2 cells, studies also found other helper T-cell pathways in different race or ethnic groups, such as Th17, Th1, and Th22 (27). For example, activation of the Th2 and Th17 pathways has been identified in the Asian atopic dermatitis population, whereas the Th2 pathway is the most common immune response in European atopic dermatitis patients (27). It is well known that T cells can cause acute and chronic kidney diseases, especially immune-mediated renal disease, which plays an important role in maintaining renal dynamic balance and repairing renal injury (28). Specifically, activation of the Th2 and T follicular helper cells promotes renal fibrosis under various stimulation by leveraging oxidative phosphorylation (OXPHOS) and glycolysis metabolism predominantly (29, 30). Activated Th17 cells exert a pro-fibrosis effect by exploiting glycolysis, OXPHOS, and fatty acid synthesis (29, 30). In addition, several bioactive components, such as reactive oxygen species and inflammatory cytokines from the autoimmune inflammatory process may impair endothelial function and consequently cause kidney vasculature damage or directly lead to kidney damage (31, 32).

Skin disease, including atopic dermatitis, is a common comorbidity in patients with CKD (7). Several case–control studies documented that xerosis caused by atopic dermatitis and other skin disease was the most common dermatological manifestation in CKD patients with and without dialysis (8, 33). In our MR study, the impact of CKD on atopic dermatitis risk was confirmed. Several potential hypotheses have been formulated to explain the increased risk of atopic dermatitis in CKD patients, including endogenous opioid system imbalance, alternation of immune status, neuropathy, functional and structural changes in the brain, and parathyroid hormone abnormalities (6). In addition, pruritus caused by atopic dermatitis and other systemic diseases has a significant effect on the patient’s quality of life (6). More focus on dermatological issue management among CKD patients is needed.

There are several strengths of the present MR study. The major merit is the MR design, which strengthened the causal inference in the bidirectional association between atopic dermatitis and CKD. This study used data from consortia with large sample sizes. Thus, our study might have adequate power to detect weak associations. In addition, we replicated the associations in two sources with independent populations and performed several sensitivity analyses. The high consistency of estimates in the two data and sensitivity analyses ensured the robustness of our findings.

Several limitations need consideration when interpreting our findings. We used data from genome-wide association analysis on CKD based on participants of multi-ancestries to enlarge statistical power. Thus, the population structure bias was likely to be introduced and bias the observed associations even though the proportion of non-European population is small, and the associations persisted in the analysis confined to individuals of the FinnGen consortium. Besides, only using the GFR to identify CKD cases in the CKDGen consortium may underestimate CKD since not all patients would develop every form of the disease or progress, and dysfunction of the glomerular barrier (represented by albuminuria) and reduced kidney function (represented by eGFR) can develop independently (34). Also, since the original GWAS for atopic dermatitis did not conduct stratified analysis across age groups, we are unable to evaluate the effect of early-onset or late-onset atopic dermatitis on CKD risk. Future atopic dermatitis GWASs based on different age groups are needed to address this. Pleiotropy is an important limitation of any MR study (35). Even though we did not detect any indication of pleiotropic effects in MR-Egger analysis, we could not rule out the possibility that the genetic instruments that we selected for atopic dermatitis and CKD might be associated with other traits that influence the risk of our target outcomes. In addition, the vertical pleiotropy in this study might exist although it is unlikely to bias causal inference in MR study (35). In detail, genetic instruments used might reflect the liability to the exposures as well as the use of biological medications for these diseases, such as drugs for moderate-to-severe skin conditions and angiotensin-converting enzyme inhibitors for CKD. In terms of time order, these pleiotropic effects related to medications are more likely to mediate the associations. In addition, the positive association between atopic dermatitis and CKD might be caused by shared genes but not a causal link. However, a possible inverse genetic correlation between two diseases minimized the bias.

## Conclusion

In conclusion, this MR study suggests a bidirectional association between atopic dermatitis and CKD. Our findings may shed a light on CKD prevention in patients with atopic dermatitis as well as dermatological issue management in CKD patients.

## Data availability statement

The original contributions presented in the study are included in the article/Supplementary material, further inquiries can be directed to the corresponding authors.

## Ethics statement

All studies included in cited genome-wide association studies had been approved by a relevant review board, and participants gave informed consent.

## Author contributions

YW, XL, and LH contributed to the study conception and design. HZ, SY, YL, and DL performed material preparation, data collection, and analysis. HZ and SY wrote the first draft of the manuscript. HZ, SY, YL, DL, ZY, LH, XL, YW, and SL commented on previous versions of the manuscript. All authors contributed to the article and approved the submitted version.

## Funding

The study is supported by research grants from the Swedish Research Council for Health, Working Life and Welfare (forte; grant no. 2018-00123) and the Swedish Research Council (Vetenskapsrådet; grant no. 2019-00977).

## Conflict of interest

The authors declare that the research was conducted in the absence of any commercial or financial relationships that could be construed as a potential conflict of interest.

## Publisher’s note

All claims expressed in this article are solely those of the authors and do not necessarily represent those of their affiliated organizations, or those of the publisher, the editors and the reviewers. Any product that may be evaluated in this article, or claim that may be made by its manufacturer, is not guaranteed or endorsed by the publisher.

## Abbreviations

CKD: chronic kidney disease
MR: Mendelian randomization
SNP: single nucleotide polymorphism
GWAS: genome-wide association study
