# Supplementary material for: Atopic dermatitis and chronic kidney disease: a bidirectional Mendelian randomization study
Source: Front Med (Lausanne). 2023 Jun 27;10:1180596. doi: 10.3389/fmed.2023.1180596 (PMC10333750; doi:10.3389/fmed.2023.1180596)
Supplement: Supplementary file 1 [file Table_1.docx]

Supporting information for

**Atopic dermatitis and chronic kidney disease: a bidirectional Mendelian randomization study**

**Supplementary Table 1**. Data used in the analysis of effect of genetic predisposition to atopic dermatitis on chronic kidney disease risk

**Supplementary Table 2**. Data used in the analysis of effect of genetic predisposition to chronic kidney disease on atopic dermatitis risk

**Supplementary Table 3**. Heterogeneity, pleiotropy and outliers in analyses

**Supplementary Table 4**. Mendelian randomization analyses for atopic dermatitis and chronic kidney disease subsets

**Supplementary Table 1**. Data used in the analysis of effect of genetic predisposition to atopic dermatitis on chronic kidney disease risk

|  |  |  |  |  | **Atopic dermatitis** | | | **Chronic kidney disease** | | |
| --- | --- | --- | --- | --- | --- | --- | --- | --- | --- | --- |
| **Source** | **SNP** | **EA** | **NEA** | **EAF** | **Beta** | **SE** | **P** | **Beta** | **SE** | **P** |
| Consortium | rs12730935 | A | G | 0.39 | 0.077 | 0.014 | 6.10E-11 | -0.012 | 0.009 | 0.194 |
| Consortium | rs61813875 | G | C | 0.02 | 0.476 | 0.043 | 5.60E-29 | 0.066 | 0.038 | 0.082 |
| Consortium | rs7512552 | T | C | 0.49 | -0.073 | 0.011 | 9.10E-10 | -0.011 | 0.008 | 0.164 |
| Consortium | rs10199605 | A | G | 0.30 | -0.073 | 0.017 | 3.40E-08 | 0.011 | 0.009 | 0.202 |
| Consortium | rs1057258 | T | C | 0.18 | -0.062 | 0.011 | 1.70E-10 | 0.005 | 0.010 | 0.642 |
| Consortium | rs112111458 | G | A | 0.13 | -0.062 | 0.011 | 9.40E-09 | -0.001 | 0.012 | 0.903 |
| Consortium | rs6419573 | T | C | 0.26 | 0.104 | 0.014 | 1.50E-13 | -0.004 | 0.010 | 0.709 |
| Consortium | rs6827756 | T | C | 0.37 | 0.077 | 0.014 | 4.20E-09 | -0.003 | 0.008 | 0.696 |
| Consortium | rs10214237 | C | T | 0.27 | -0.073 | 0.017 | 2.90E-08 | -0.006 | 0.009 | 0.485 |
| Consortium | rs12188917 | C | T | 0.21 | 0.131 | 0.018 | 4.00E-17 | -0.015 | 0.011 | 0.181 |
| Consortium | rs4713555 | T | G | 0.27 | -0.094 | 0.011 | 5.40E-09 | -0.007 | 0.008 | 0.386 |
| Consortium | rs6473227 | A | C | 0.61 | -0.073 | 0.011 | 1.40E-09 | -0.017 | 0.008 | 0.029 |
| Consortium | rs6602364 | G | C | 0.45 | 0.077 | 0.014 | 1.50E-09 | -0.001 | 0.008 | 0.941 |
| Consortium | rs10791824 | G | A | 0.57 | 0.113 | 0.014 | 2.10E-19 | 0.028 | 0.008 | 0.000 |
| Consortium | rs2212434 | T | C | 0.45 | 0.086 | 0.009 | 4.60E-13 | 0.016 | 0.008 | 0.045 |
| Consortium | rs7127307 | C | T | 0.47 | -0.073 | 0.017 | 3.90E-10 | -0.012 | 0.008 | 0.116 |
| Consortium | rs2227483 | A | T | 0.44 | -0.062 | 0.005 | 6.70E-16 | 0.005 | 0.008 | 0.521 |
| Consortium | rs2038255 | T | C | 0.18 | 0.104 | 0.019 | 1.80E-10 | -0.007 | 0.010 | 0.494 |
| Consortium | rs2041733 | C | T | 0.55 | -0.083 | 0.011 | 2.50E-11 | -0.007 | 0.008 | 0.400 |
| Consortium | rs2918307 | G | A | 0.16 | 0.113 | 0.019 | 4.60E-12 | -0.006 | 0.012 | 0.620 |
| Consortium | rs4809219 | C | A | 0.27 | -0.105 | 0.017 | 7.00E-13 | -0.018 | 0.009 | 0.049 |
| FinnGen | rs12730935 | A | G | 0.39 | 0.077 | 0.014 | 6.10E-11 | 0.004 | 0.026 | 0.892 |
| FinnGen | rs61813875 | G | C | 0.02 | 0.476 | 0.043 | 5.60E-29 | -0.115 | 0.129 | 0.373 |
| FinnGen | rs7512552 | T | C | 0.49 | -0.073 | 0.011 | 9.10E-10 | 0.016 | 0.024 | 0.490 |
| FinnGen | rs10199605 | A | G | 0.30 | -0.073 | 0.017 | 3.40E-08 | -0.015 | 0.024 | 0.526 |
| FinnGen | rs1057258 | T | C | 0.18 | -0.062 | 0.011 | 1.70E-10 | 0.004 | 0.034 | 0.903 |
| FinnGen | rs112111458 | G | A | 0.13 | -0.062 | 0.011 | 9.40E-09 | 0.011 | 0.046 | 0.805 |
| FinnGen | rs6419573 | T | C | 0.26 | 0.104 | 0.014 | 1.50E-13 | -0.041 | 0.030 | 0.168 |
| FinnGen | rs6827756 | T | C | 0.37 | 0.077 | 0.014 | 4.20E-09 | 0.010 | 0.024 | 0.675 |
| FinnGen | rs10214237 | C | T | 0.27 | -0.073 | 0.017 | 2.90E-08 | -0.020 | 0.025 | 0.426 |
| FinnGen | rs12188917 | C | T | 0.21 | 0.131 | 0.018 | 4.00E-17 | 0.015 | 0.027 | 0.588 |
| FinnGen | rs4713555 | T | G | 0.27 | -0.094 | 0.011 | 5.40E-09 | -0.091 | 0.027 | 0.001 |
| FinnGen | rs6473227 | A | C | 0.61 | -0.073 | 0.011 | 1.40E-09 | 0.003 | 0.025 | 0.912 |
| FinnGen | rs6602364 | G | C | 0.45 | 0.077 | 0.014 | 1.50E-09 | 0.023 | 0.024 | 0.338 |
| FinnGen | rs10791824 | G | A | 0.57 | 0.113 | 0.014 | 2.10E-19 | 0.034 | 0.025 | 0.172 |
| FinnGen | rs2212434 | T | C | 0.45 | 0.086 | 0.009 | 4.60E-13 | 0.027 | 0.024 | 0.262 |
| FinnGen | rs7127307 | C | T | 0.47 | -0.073 | 0.017 | 3.90E-10 | -0.044 | 0.024 | 0.067 |
| FinnGen | rs2227483 | A | T | 0.44 | -0.062 | 0.005 | 6.70E-16 | 0.034 | 0.024 | 0.158 |
| FinnGen | rs2038255 | T | C | 0.18 | 0.104 | 0.019 | 1.80E-10 | -0.022 | 0.029 | 0.456 |
| FinnGen | rs2041733 | C | T | 0.55 | -0.083 | 0.011 | 2.50E-11 | 0.027 | 0.024 | 0.249 |
| FinnGen | rs2918307 | G | A | 0.16 | 0.113 | 0.019 | 4.60E-12 | -0.027 | 0.029 | 0.347 |
| FinnGen | rs4809219 | C | A | 0.27 | -0.105 | 0.017 | 7.00E-13 | -0.041 | 0.030 | 0.171 |

EA, effect allele; EAF, effect allele frequency; NEA, non-effect allele; SE, standard error; SNP, single nucleotide polymorphism.

**Supplementary Table 2**. Data used in the analysis of effect of genetic predisposition to chronic kidney disease on atopic dermatitis risk

|  |  |  |  |  | **Chronic kidney disease** | | | **Atopic dermatitis** | | |
| --- | --- | --- | --- | --- | --- | --- | --- | --- | --- | --- |
| **Source** | **SNP** | **EA** | **NEA** | **EA** | **Beta** | **SE** | **P** | **Beta** | **SE** | **P** |
| Consortium | rs2484640 | T | G | 0.54 | -0.070 | 0.008 | 2.50E-18 | -0.019 | 0.017 | 0.258 |
| Consortium | rs1015784 | C | G | 0.51 | 0.043 | 0.008 | 3.20E-08 | -0.017 | 0.017 | 0.321 |
| Consortium | rs1047891 | A | C | 0.28 | 0.052 | 0.009 | 2.99E-08 | -0.011 | 0.019 | 0.562 |
| Consortium | rs11123169 | T | C | 0.68 | -0.053 | 0.008 | 2.31E-10 | -0.025 | 0.018 | 0.166 |
| Consortium | rs13389381 | T | C | 0.89 | -0.083 | 0.015 | 3.98E-08 | -0.007 | 0.034 | 0.833 |
| Consortium | rs187355703 | C | G | 0.98 | -0.195 | 0.031 | 2.78E-10 | -0.056 | 0.056 | 0.318 |
| Consortium | rs1881245 | A | G | 0.26 | -0.057 | 0.010 | 2.28E-08 | 0.007 | 0.020 | 0.713 |
| Consortium | rs12509595 | T | C | 0.69 | 0.067 | 0.009 | 5.08E-14 | -0.006 | 0.019 | 0.739 |
| Consortium | rs13146355 | A | G | 0.39 | 0.068 | 0.008 | 9.27E-17 | -0.007 | 0.017 | 0.685 |
| Consortium | rs35716097 | T | C | 0.33 | 0.065 | 0.009 | 1.73E-13 | 0.060 | 0.020 | 0.002 |
| Consortium | rs835223 | T | C | 0.37 | 0.060 | 0.009 | 1.02E-11 | 0.011 | 0.017 | 0.518 |
| Consortium | rs12207180 | A | T | 0.11 | 0.084 | 0.013 | 1.10E-10 | 0.017 | 0.026 | 0.520 |
| Consortium | rs6929219 | T | C | 0.92 | 0.092 | 0.016 | 2.81E-09 | -0.031 | 0.032 | 0.333 |
| Consortium | rs881858 | A | G | 0.72 | 0.059 | 0.009 | 6.39E-11 | 0.040 | 0.019 | 0.037 |
| Consortium | rs9474801 | A | G | 0.31 | 0.049 | 0.009 | 7.31E-09 | 0.014 | 0.018 | 0.440 |
| Consortium | rs13230625 | A | G | 0.58 | 0.066 | 0.009 | 8.51E-13 | -0.021 | 0.026 | 0.417 |
| Consortium | rs7805747 | A | G | 0.27 | 0.105 | 0.010 | 4.52E-26 | -0.009 | 0.020 | 0.653 |
| Consortium | rs868822 | T | G | 0.35 | -0.047 | 0.009 | 2.65E-08 | 0.024 | 0.019 | 0.197 |
| Consortium | rs1705696 | A | T | 0.55 | 0.044 | 0.008 | 1.59E-08 | -0.017 | 0.017 | 0.325 |
| Consortium | rs1412987 | A | G | 0.68 | -0.062 | 0.010 | 6.90E-10 | -0.021 | 0.020 | 0.276 |
| Consortium | rs7908590 | C | G | 0.93 | -0.130 | 0.019 | 2.41E-12 | -0.083 | 0.035 | 0.018 |
| Consortium | rs10896037 | A | G | 0.69 | -0.067 | 0.009 | 4.72E-13 | -0.118 | 0.017 | 0.000 |
| Consortium | rs2293579 | A | G | 0.35 | 0.046 | 0.008 | 1.12E-08 | 0.018 | 0.017 | 0.302 |
| Consortium | rs963837 | T | C | 0.59 | 0.081 | 0.008 | 1.58E-21 | 0.003 | 0.017 | 0.847 |
| Consortium | rs17730281 | A | G | 0.28 | -0.079 | 0.009 | 1.42E-18 | 0.063 | 0.020 | 0.002 |
| Consortium | rs2453533 | A | C | 0.43 | 0.074 | 0.009 | 8.57E-17 | 0.010 | 0.017 | 0.560 |
| Consortium | rs4886747 | A | G | 0.81 | 0.059 | 0.011 | 4.99E-08 | -0.030 | 0.023 | 0.183 |
| Consortium | rs8026369 | A | G | 0.38 | -0.056 | 0.008 | 5.14E-12 | 0.010 | 0.017 | 0.570 |
| Consortium | rs6497474 | T | C | 0.42 | 0.086 | 0.013 | 4.26E-11 | 0.087 | 0.040 | 0.030 |
| Consortium | rs7195855 | T | C | 0.71 | -0.090 | 0.012 | 3.55E-13 | -0.017 | 0.034 | 0.615 |
| Consortium | rs77924615 | A | G | 0.20 | -0.209 | 0.011 | 0.00E+00 | -0.009 | 0.024 | 0.702 |
| Consortium | rs78751740 | A | G | 0.13 | -0.141 | 0.020 | 1.38E-12 | -0.021 | 0.083 | 0.799 |
| Consortium | rs2285639 | T | C | 0.42 | -0.047 | 0.008 | 2.67E-09 | 0.001 | 0.017 | 0.935 |
| Consortium | rs76578497 | T | G | 0.98 | -0.212 | 0.036 | 2.98E-09 | -0.176 | 0.112 | 0.115 |
| Consortium | rs16942751 | A | C | 0.18 | 0.073 | 0.012 | 2.06E-10 | 0.022 | 0.030 | 0.463 |
| Consortium | rs8096658 | C | G | 0.56 | -0.064 | 0.009 | 2.02E-12 | -0.020 | 0.021 | 0.349 |
| Consortium | rs2823140 | A | G | 0.33 | 0.050 | 0.009 | 8.47E-09 | 0.025 | 0.018 | 0.150 |
| FinnGen | rs2484640 | T | G | 0.54 | -0.070 | 0.008 | 2.50E-18 | 0.002 | 0.018 | 0.932 |
| FinnGen | rs1015784 | C | G | 0.51 | 0.043 | 0.008 | 3.20E-08 | 0.029 | 0.018 | 0.111 |
| FinnGen | rs1047891 | A | C | 0.28 | 0.052 | 0.009 | 2.99E-08 | 0.045 | 0.019 | 0.020 |
| FinnGen | rs11123169 | T | C | 0.68 | -0.053 | 0.008 | 2.31E-10 | -0.018 | 0.019 | 0.348 |
| FinnGen | rs13389381 | T | C | 0.89 | -0.083 | 0.015 | 3.98E-08 | -0.044 | 0.048 | 0.354 |
| FinnGen | rs187355703 | C | G | 0.98 | -0.195 | 0.031 | 2.78E-10 | 0.021 | 0.060 | 0.727 |
| FinnGen | rs1881245 | A | G | 0.26 | -0.057 | 0.010 | 2.28E-08 | 0.015 | 0.022 | 0.498 |
| FinnGen | rs12509595 | T | C | 0.69 | 0.067 | 0.009 | 5.08E-14 | 0.036 | 0.020 | 0.069 |
| FinnGen | rs13146355 | A | G | 0.39 | 0.068 | 0.008 | 9.27E-17 | 0.007 | 0.018 | 0.696 |
| FinnGen | rs35716097 | T | C | 0.33 | 0.065 | 0.009 | 1.73E-13 | 0.026 | 0.019 | 0.164 |
| FinnGen | rs835223 | T | C | 0.37 | 0.060 | 0.009 | 1.02E-11 | -0.023 | 0.019 | 0.222 |
| FinnGen | rs12207180 | A | T | 0.11 | 0.084 | 0.013 | 1.10E-10 | -0.041 | 0.029 | 0.159 |
| FinnGen | rs6929219 | T | C | 0.92 | 0.092 | 0.016 | 2.81E-09 | 0.027 | 0.036 | 0.447 |
| FinnGen | rs881858 | A | G | 0.72 | 0.059 | 0.009 | 6.39E-11 | 0.009 | 0.020 | 0.637 |
| FinnGen | rs9474801 | A | G | 0.31 | 0.049 | 0.009 | 7.31E-09 | 0.029 | 0.021 | 0.152 |
| FinnGen | rs13230625 | A | G | 0.58 | 0.066 | 0.009 | 8.51E-13 | 0.015 | 0.019 | 0.431 |
| FinnGen | rs7805747 | A | G | 0.27 | 0.105 | 0.010 | 4.52E-26 | -0.014 | 0.023 | 0.551 |
| FinnGen | rs868822 | T | G | 0.35 | -0.047 | 0.009 | 2.65E-08 | 0.011 | 0.020 | 0.606 |
| FinnGen | rs1705696 | A | T | 0.55 | 0.044 | 0.008 | 1.59E-08 | -0.006 | 0.019 | 0.758 |
| FinnGen | rs1412987 | A | G | 0.68 | -0.062 | 0.010 | 6.90E-10 | -0.010 | 0.021 | 0.644 |
| FinnGen | rs7908590 | C | G | 0.93 | -0.130 | 0.019 | 2.41E-12 | -0.037 | 0.046 | 0.420 |
| FinnGen | rs10896037 | A | G | 0.69 | -0.067 | 0.009 | 4.72E-13 | -0.127 | 0.018 | 0.000 |
| FinnGen | rs2293579 | A | G | 0.35 | 0.046 | 0.008 | 1.12E-08 | -0.030 | 0.020 | 0.137 |
| FinnGen | rs963837 | T | C | 0.59 | 0.081 | 0.008 | 1.58E-21 | 0.001 | 0.018 | 0.965 |
| FinnGen | rs17730281 | A | G | 0.28 | -0.079 | 0.009 | 1.42E-18 | 0.033 | 0.021 | 0.113 |
| FinnGen | rs2453533 | A | C | 0.43 | 0.074 | 0.009 | 8.57E-17 | 0.008 | 0.018 | 0.662 |
| FinnGen | rs4886747 | A | G | 0.81 | 0.059 | 0.011 | 4.99E-08 | 0.020 | 0.023 | 0.383 |
| FinnGen | rs8026369 | A | G | 0.38 | -0.056 | 0.008 | 5.14E-12 | -0.010 | 0.019 | 0.613 |
| FinnGen | rs6497474 | T | C | 0.42 | 0.086 | 0.013 | 4.26E-11 | 0.004 | 0.033 | 0.908 |
| FinnGen | rs7195855 | T | C | 0.71 | -0.090 | 0.012 | 3.55E-13 | 0.025 | 0.038 | 0.509 |
| FinnGen | rs77924615 | A | G | 0.20 | -0.209 | 0.011 | 6.66E-86 | -0.027 | 0.022 | 0.218 |
| FinnGen | rs78751740 | A | G | 0.13 | -0.141 | 0.020 | 1.38E-12 | 0.042 | 0.065 | 0.518 |
| FinnGen | rs2285639 | T | C | 0.42 | -0.047 | 0.008 | 2.67E-09 | 0.011 | 0.019 | 0.568 |
| FinnGen | rs76578497 | T | G | 0.98 | -0.212 | 0.036 | 2.98E-09 | -0.129 | 0.084 | 0.125 |
| FinnGen | rs16942751 | A | C | 0.18 | 0.073 | 0.012 | 2.06E-10 | -0.030 | 0.028 | 0.278 |
| FinnGen | rs8096658 | C | G | 0.56 | -0.064 | 0.009 | 2.02E-12 | -0.004 | 0.018 | 0.837 |
| FinnGen | rs2823140 | A | G | 0.33 | 0.050 | 0.009 | 8.47E-09 | -0.020 | 0.019 | 0.287 |

EA, effect allele; EAF, effect allele frequency; NEA, non-effect allele; SE, standard error; SNP, single nucleotide polymorphism.

**Supplementary Table 3**. Heterogeneity, pleiotropy and outliers in analyses

| **Exposure & source** | **SNPs used** | **Cochrane's Q** | **Intercept** | ***p* for intercept** | **Outliers** |
| --- | --- | --- | --- | --- | --- |
| **Atopic dermatitis** |  |  |  |  |  |
| CKDGen consortium | 21 | 34 | -0.01 | 0.45 | 1 |
| FinnGen consortium | 21 | 29 | 0.01 | 0.66 | 1 |
| **Chronic kidney disease** |  |  |  |  |  |
| EAGLE consortium | 37 | 94 | 0.00 | 0.94 | 2 |
| FinnGen consortium | 37 | 80 | 0.00 | 0.79 | 1 |

EAGLE, EArly Genetics and Lifecourse Epidemiology; SNPs, single nucleotide polymorphism.

**Supplementary Table 4**. Mendelian randomization analyses for atopic dermatitis and chronic kidney disease subsets

| **Outcome** | **Data Source** | **N_SNP** | **Method** | **Beta** | **SE** | **P_effect** | **P_heterogeneity** | **P_intercept** | **Outlier** |
| --- | --- | --- | --- | --- | --- | --- | --- | --- | --- |
| membranous nephropathy | Available GWAS | 16 | IVW | 0.479 | 0.420 | 0.253 | <0.001 |  |  |
|  |  |  | Weighted median | 0.223 | 0.191 | 0.243 |  |  |  |
|  |  |  | MR-Egger | 1.215 | 2.347 | 0.613 |  | 0.755 |  |
|  |  |  | MR-PRESSO | 0.126 | 0.121 | 0.314 |  |  | 1 |
| Diabetic nephropathy | FinnGen | 20 | IVW | 0.161 | 0.118 | 0.173 | <0.001 |  |  |
|  |  |  | Weighted median | 0.051 | 0.110 | 0.641 |  |  |  |
|  |  |  | MR-Egger | 0.172 | 0.423 | 0.690 |  | 0.978 |  |
|  |  |  | MR-PRESSO | 0.075 | 0.089 | 0.414 |  |  | 1 |
| Glomerular nephritis | FinnGen | 20 | IVW | 0.047 | 0.082 | 0.562 | 0.019 |  |  |
|  |  |  | Weighted median | 0.027 | 0.092 | 0.766 |  |  |  |
|  |  |  | MR-Egger | -0.070 | 0.290 | 0.813 |  | 0.679 |  |
|  |  |  | MR-PRESSO | -0.004 | 0.069 | 0.958 |  |  | 1 |
| Hypertensive nephropathy | FinnGen | 20 | IVW | -0.225 | 0.207 | 0.277 | 0.221 |  |  |
|  |  |  | Weighted median | -0.104 | 0.276 | 0.707 |  |  |  |
|  |  |  | MR-Egger | 0.305 | 0.729 | 0.681 |  | 0.458 |  |
|  |  |  | MR-PRESSO | NA | NA | NA |  |  | 0 |
| Nephrotic syndrome | FinnGen | 20 | IVW | 0.378 | 0.189 | 0.046 | 0.378 |  |  |
|  |  |  | Weighted median | 0.326 | 0.264 | 0.217 |  |  |  |

N_SNP, the number of SNPs used in the analysis. IVW, inverse variance weighted method.
